# Direct microcontact printing of oligonucleotides for biochip applications

**DOI:** 10.1186/1477-3155-3-7

**Published:** 2005-07-01

**Authors:** C Thibault, V Le Berre, S Casimirius, E Trévisiol, J François, C Vieu

**Affiliations:** 1 LAAS-CNRS, 7, avenue du Colonel Roche 31077 TOULOUSE Cedex 4; 2 Biochips Platform Genopole Toulouse, UMR-CNRS 5504 & INRA 792, 135, avenue de Rangueil, 31077 TOULOUSE Cedex 4; 3 Laboratoire de Biotechnologie & Bioprocédés, UMR-CNRS 5504 & INRA 792, 135, avenue de Rangueil, 31077 TOULOUSE Cedex 4

**Keywords:** microcontact printing, elastomeric stamp, DNA immobilisation, biochips, detection of mutations

## Abstract

**Background:**

A critical step in the fabrication of biochips is the controlled placement of probes molecules on solid surfaces. This is currently performed by sequential deposition of probes on a target surface with split or solid pins. In this article, we present a cost-effective procedure namely microcontact printing using stamps, for a parallel deposition of probes applicable for manufacturing biochips.

**Results:**

Contrary to a previous work, we showed that the stamps tailored with an elastomeric poly(dimethylsiloxane) material did not require any surface modification to be able to adsorb oligonucleotides or PCR products. The adsorbed DNA molecules are subsequently printed efficiently on a target surface with high sub-micron resolution. Secondly, we showed that successive stamping is characterized by an exponential decay of the amount of transferred DNA molecules to the surface up the 4^th ^print, then followed by a second regime of transfer that was dependent on the contact time and which resulted in reduced quality of the features. Thus, while consecutive stamping was possible, this procedure turned out to be less reproducible and more time consuming than simply re-inking the stamps between each print. Thirdly, we showed that the hybridization signals on arrays made by microcontact printing were 5 to 10-times higher than those made by conventional spotting methods. Finally, we demonstrated the validity of this microcontact printing method in manufacturing oligonucleotides arrays for mutations recognition in a yeast gene.

**Conclusion:**

The microcontact printing can be considered as a new potential technology platform to pattern DNA microarrays that may have significant advantages over the conventional spotting technologies as it is easy to implement, it uses low cost material to make the stamp, and the arrays made by this technology are 10-times more sensitive in term of hybridization signals than those manufactured by conventional spotting technology.

## Background

DNA microarrays have rapidly evolved to become one of the essential tools to investigate expression or mutation of thousands of genes simultaneously. Two main technology platforms for manufacturing DNA chips have emerged. The first platform uses the immobilization of prefabricated DNA or oligonucleotides by spotting on functionalized glass slides using metal pins as originally developed by Brown and collaborators (see ), or by a non-contact method using piezoelectric liquid handling [1]. The second platform rests on the direct *in-situ *synthesis of oligonucleotides (between 20 to 70 mers in general) on glass slides or silicon surfaces, as developed by Affymetrix or Agilent [2]. A typical characteristic of these techniques is the sequential nature of the process. One molecule is deposited after another or one base is added to the previous one, with the consequence that each array is made as an original with a reduced throughput, although Affymetrix microarrays manufacturing involves combinatorial processes that allow multiple microarrays (around 96) to be synthesized in parallel in matters of hours. Nevertheless, these technology platforms needs sophisticated equipment, leading to high density arrays that can be too expensive for production and utilization of simple-customized-DNA arrays.

There is a need for alternative patterning methods that must be very simple, reproducible, cost-effective, and eventually transferable to any laboratories for their own problematic. The microcontact printing (μCP) could fulfill this requirement as it is a printing technology that uses cheap elastomeric stamps made usually of polydimethylsiloxane (PDMS) and which exhibits relief patterns at the micron and nanoscale [3]. These stamps let to parallel deposition of molecules on a target surface, in the same manner as the printing of a page of book instead of a letter being written individually to compose the page. Previous works demonstrated that proteins can be deposited on a substrate surface by microcontact printing (μCP) [4,5]. More recently, Lange *et al*. [6] showed that μCP technique can be used to deposit DNA molecules with a PDMS surface of the stamp chemically modified to enable the DNA molecules to stick on the stamp. This functionalization step strongly restricted the speed of this technology, as it takes several hours from the conversion of the CH_3 _terminated surface of the PDMS into an aminated surface to complete inking of the stamps prior to printing the target surface.

**Figure 1:**
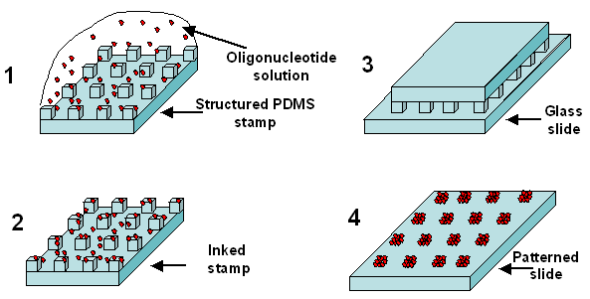
**Principe of microcontact printing of DNA molecules**. (1) Inking of the stamp with the oligonucleotide solution, a 1 cm^2 ^stamp is loaded with a 2 to 20 μl droplet of solution for a given time (2) drying of the stamp under Nitrogen stream, (3) manual contact between the inked PDMS stamp and the glass slide, (4) probe molecules are transferred on the slide along patterns that correspond to the relief structures of the PDMS stamp.

In this paper, we demonstrate that μCP can be used to fabricate DNA biochips directly without any surface modification of the stamps. We show that inking and contact times of less than 30 seconds give high quality and high resolution arrays by μCP. According to our new variant of the process, the stamp is simply inked with the molecules of interest, dried under a nitrogen stream and then printed manually onto the substrate surface (see Fig. 1). It is foreseen that this technology platform will be highly competitive for high throughput analysis of gene expression and mutation detection analyses. Moreover, this technique can be easily implemented for sub-micron patterns as demonstrated previously [6] and in this work.

## Results and Discussion

The two main steps of μCP are the adsorption of the biomolecules on the stamp (inking process) and the transfer from the stamp to a target surface (contact printing). It is important that the retention of molecules on the stamp surface does not prevent their subsequent transfer to the slide, and that the inking and the contact time were as short as possible for optimizing the high throughput of the technique. In a recent work [6], this compromise was obtained by a specific chemical treatment of the elastomeric poly(dimethylsiloxane) material (PDMS) of the stamp after molding. In contrast to this report, we found that untreated PDMS stamp that has a strong hydrophobic surface after curing, easily adsorbs a sufficient amount of DNA molecules within few seconds while allowing their subsequent deposition by contact on microscope glass slides or silicon. The printing process works for untreated glass or silicon surfaces, but real bioassays were carried out on treated glass surfaces enabling strong binding of the probe molecules. During the contact, the purpose is to transfer efficiently and as quick as possible the molecules from the stamp surface to the slide without affecting the size of the patterns. A specific chemistry on the surface of the slide is also important for the attachment of the probes after taking away the stamp from the surface. We also verified that stamps could be reused several times after cleaning in deionized water. The experiments detailed below aim at investigating the influence of several parameters including the surface chemistry of the slide, the inking and the contact time of the stamp, and to demonstrate the potentiality of this technique for actual biochips.

### Surface chemistry and high uniformity of DNA printing on target surfaces

**Figure 2:**
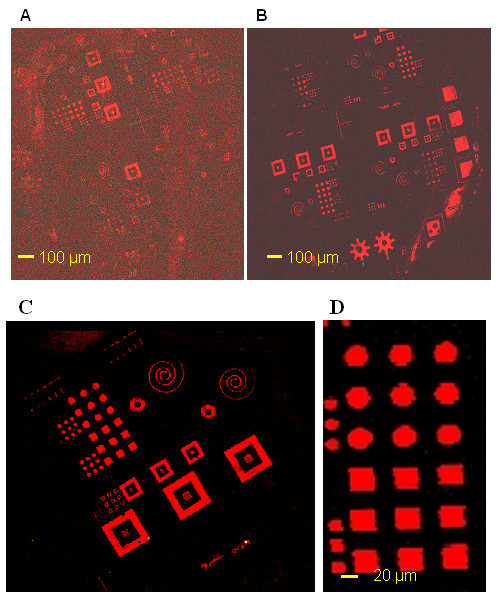
**Comparison between two types of slides**. Fluorescence images of printed micronic patterns. Stamp was incubated with a 35-mers probe oligonucleotide for 30 sec, then put in contact for 15 sec with two types of microscope glass slides. A, electrostatic slide (ultra Gap, corning), B, dendrislide (home made slide). Slides were then incubated with a 15-mer 5'-Cy5 labeled oligonucleotide. C and D are a zoom area of B.

**Figure 3:**
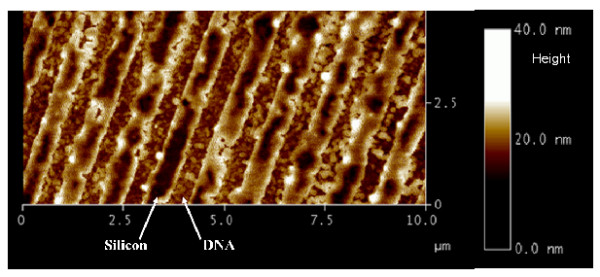
**Example of DNA printing at the submicronic scale**. AFM image (taping mode) of 30-mers 5'-GCATGCTTAGTTGCTATTATCAAAATA-3', corresponding to *BCK2 *yeast gene printed on an untreated silicon surface. The pitch of the periodic array of lines is 1 μm. Note that the chemical surface states of the silicon was not really controlled: rough native oxide.

Experiments reported in this paper were carried out using two different type of glass slides that differed by their surface functionalization: positively charged amine glass slides (Ultra Gap, Dow corning) and dendrislides, which are glass slides that have been functionalized with nanometric spherical dendrimeric particles bearing aldehydes reactive group at the periphery for covalent attachment of the 5'-NH_2 _probes [7,8]. These two types of functionalized slides were printed for 15 sec with a stamp that has been incubated for 30 sec with a 10 μM solution of 35-mers 5'-NH_2 _probe in Na-phosphate buffer at pH 9.0. Hybridisation was achieved using a 15-mer 5'Cy5 target complementary to the 35-mer 5'-NH_2 _probe. As shown on Fig. 2, the micronic features of the stamp (squares, disks, gears, crosses, spirals, ...) were clearly noticeable on both types of glass slides. However, we observed systematically a greater signal to noise ratio, a better uniformity and edge definition of the spots with dendrislides (Fig. 2B) than with electrostatic slides (Figure 3A). This result is consistent with our previous report that the functionalization of surface with dendrimers reduces the non specific adsorption of fluorescent material [8]. In addition, the "donut" formation of spots frequently obtained after deposition of DNA molecules by contact spotting was no longer observed since the μCP is a "dry" deposition technique. This enables a better treatment of the fluorescence images for quantitative analysis. The upper part of Fig. 2C shows few lines on the array that exhibit a pitch of 4 μm which could only be seen as very small red spots because the fluorescent scanner cannot resolve the features. A magnification on conventional features (*i.e. *squares and disks) is shown in Fig. 2D. On this image, the contour of the patterns was mainly blurred by the pixel size of the scanner. In order to allow Atomic Force Microscopy (AFM) characterization, submicronic features were printed on silicon surface instead of glass slides to minimize the surface roughness. These patterns consisted in a periodic array of 500 nm wide lines at a pitch of 1 μm. As shown in Fig. 3, the 500 nm wide lines are clearly visible and the printed oligonucleotides appear as small aggregates that could be distinguished from the smooth surface of the silicon substrate. It is worth noticing that in this case the surface of the sample could not be rinsed after printing, because the untreated silicon surface does not provide strong adhesion of DNA molecules. Edge roughness and small aggregates visible on the image can be possibly attributed to residues coming from the buffer solution.

### Inking time

In our first trial, the molded PDMS stamps were incubated at room temperature in the oligonucleotides solution for different times ranging from 30 sec to 1 hr, and then printed on a dendrislide after drying. Under these conditions, a very high and saturating fluorescent intensity was obtained independently of the inking time, likely because the amount of transferred fluorescent DNA molecules to the surface was already very high at the shortest inking time tested. It was even possible to observe deleterious effects for excessive inking times due to excess fluorescent material deposited at the periphery of the stamp (data not shown). These results indicated that the PDMS surface was saturated with DNA molecules in less than 30 sec of inking. We therefore reduced the inking time to a period that is easily compatible with a handling procedure of the stamps, *i.e. *15 sec.

**Figure 4:**
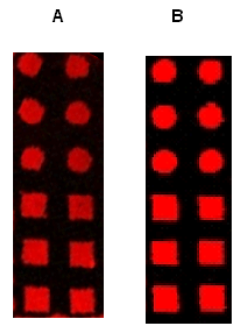
**Comparison between oligonucleotides and PCR fragments**. Fluorescent images of typical micrometric printed features. Stamp was incubated for 30 sec with a 500 bp PCR fragment (dsDNA) of the yeast *HSP12 *gene (A) or with a 20-mer oligonucleotide of the same yeast gene (B), then set in contact manually for 15 sec with a dendrislide. Hybridisation was carried out with *HSP12 *complementary Cy5-labelled oligonucleotide. Values of fluorescence intensity were measured at 635 nm with the GenePix 4000B from axon at 600 PMT. Mean intensity at 635 of 12 features on two experiments – Background was 2120 for A and 4119 for B.

To explain the excellent performance of this technique to print DNA probes, we suggest that a hydrophobic interaction takes place between the PDMS surface of the stamp and single strand DNA molecules, since the PDMS surface is highly hydrophobic, and the DNA strand can also exhibit hydrophobic properties through its bases content, even though it is an hydrophilic molecule. Moreover, hydrophobic interactions are 10 to 100 times stronger and have a longer range of action than the Van der Waals interactions [9,10]. On the other hand, a fast and efficient transfer of the DNA probes from the stamp to the slide required that the interacting forces between the oligonucleotides and the PDMS surface must be weaker than those occurring between the oligonucleotides and the surface of the slide. This was verified in our experiments for both positively charged and hydrophobic dendrimeric activated surface slides. As a consequence, preserving the hydrophobicity of the PDMS stamp is clearly a key point in order to reduce the inking times for DNA printing and to favor the subsequent transfer of the molecules to either a positive charged or a hydrophobic surface. This is the main difference between our work and that of Lange *et al *[6]. In this latter work, the adsorption of DNA probes on the stamp was mainly based on electrostatic interactions with the consequence of long inking period (45 min.). In addition, as the surface treatment of PDMS is known to be unstable on air, our process, which does not involve any surface modification after molding, should be more reproducible and should allow the reusability of the stamp (see below). It is worth to note that similar results were obtained using long single DNA molecules or double stranded PCR fragments. However, as can be seen in Fig. 4, the signal intensity was significantly lower with stamped PCR products than with oligonucleotides. This observation was actually not specific to this technique since the same results were observed using conventional fabrication of arrays by mechanical spotting (V. Le Berre, unpublished data).

### Contact time and successive prints

**Figure 5:**
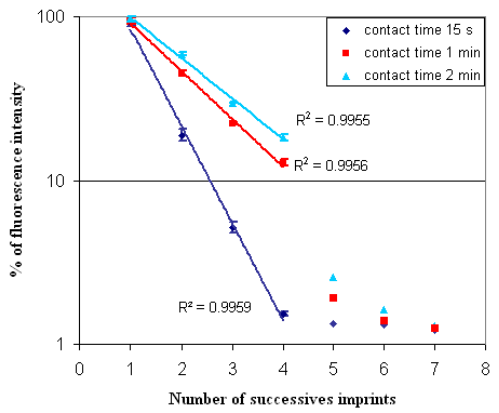
**Fluorescence signal variation for successive prints**. Variation of the fluorescence intensity for successive prints and for three different contact times (15 seconds, 1 minute and 2 minutes) between the stamp and the slide. Stamp was incubated with a 35-mer 5'-labelled Cy5 oligonucleotide for 30 sec than put in contact with the dendrislides. The value of fluorescence intensity (fluorescent – background) was measured at 635 nm with Genepix scanner under 600 PMT optical excitation. Each point represents an average of 4 independent experiments. Fittings of the data points with an exponential linear regression (solid lines), exhibits good agreement as attested by the reported correlation factors R.

To identify the transfer mechanisms of the molecules from the stamp surface to the slide, we investigated the influence of the contact time and the evolution of fluorescent signals after successive prints with the same stamp loaded with a fluorescent 35-mer 5'-labelled Cy5-oligonucleotide-3'NH_2 _(5'Cy5-TTAGCGCATTTTGGCATATTTGGGCGGACAACTT-NH_2_-3'). On the same slide, consecutive stamping steps were performed with a contact time of 15 sec, 1 min or 2 min, which took in total 2 to 20 min to pattern a dendrislide with 10 successive prints. To evaluate the change in fluorescence intensity along the successive print, the total intensity subtracted from the local background of specific features on the patterned slide were integrated and compared to the total intensity from the first print which was set arbitrarily at 100%. As shown on Fig. 5, this change followed an exponential decay up to the 4^th ^stamping, and surprisingly, this decay was dependent of the contact time. The following equation

**Figure 6:**
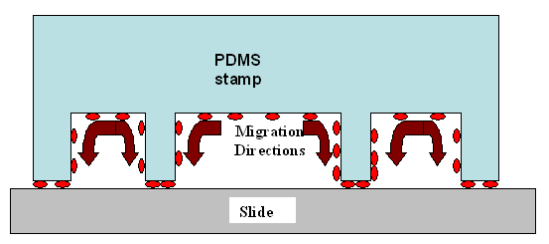
**Proposed mechanism for the diffusion of oligonucleotides during stamping**. This picture shows schematically the possible migration direction of the oligonucleotides on the stamp surface during contact. This flow could explain the preferential deposition of molecules at the rim of the patterns.

**Figure 7:**
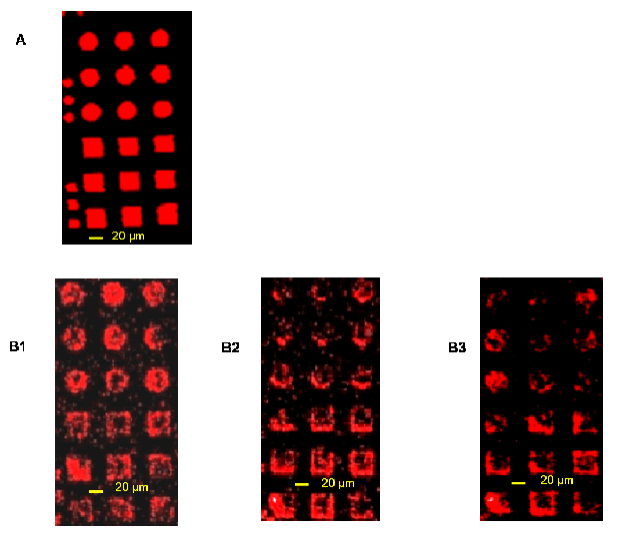
**Comparison between first and last print with the same stamp**. (A) shows the fluorescent image of the patterns transferred at the first print, and (B) shows the printing patterns after 5^th ^(B1), 6^th ^(B2) and 7^th ^print (B3). Stamps were inked with a 15-mer 5'-labelled Cy5 oligonucleotide for 30 sec and then set in contact for 2 min with the dendrislide. The well defined features is shown in (A) whereas only the rims of the patterns were detected after the 4^th ^print (B).

-dN/dn = kN

where N is the number of molecules deposited on the slide at print number n, could be used to determine the characteristic of k, a kind of sticking coefficient of the molecules on the surface. The extracted values for k turned out to be dependent upon the contact time, with k increasing as the contact time decreased (k = 1.36 for t = 15 s, k = 0.67 for t = 1 min, k = 0.57 for t = 2 min). This result indicated that longer the contact time, slower was the depletion of the stamp in biomolecules. This behavior is suggestive of a slow diffusion of the molecules retained inside the cavity of the PDMS stamp to its relief structures that are in contact with the slides, as depicted in Fig. 6. It is therefore expected to observe a slower decrease of the fluorescence intensity for increasing contact times because there is more time for the biomolecules to migrate to the surface. In addition, we calculated that the k coefficient roughly changes with the inverse of the square root of the contact time, which is consistent with a diffusion limited deposition mechanism. Accordingly, the exponential decay of the fluorescence signal was no longer valid after 4 successive printing steps (Fig. 6). For n > 4, the number of molecules initially adsorbed on the relief structures of the PDMS stamp has been largely depleted in previous prints. However, a low fluorescence intensity that decrease very slowly from the 5^th ^to the 7^th ^print was still measured. This suggested a slow diffusion of molecules from the edges of the pattern to the slides during the contact. In that case, the number of printed molecules should be higher at the periphery of the features than in the center. The fluorescence images of the 5^th ^to the 7^th ^print for a contact time of 2 min nicely confirmed this assumption (Fig. 7). Essentially the rims of the specific features were recognizable likely because the remaining molecules had enough time to migrate from the edges of the relief printing of the stamp to the glass surface during the contact time. Thus, at shorter contact times, the fluorescence images were even worse (not shown), and hence the intensity values were lower (see Fig. 5).

As a conclusion of this section, we clearly identified some problems related to diffusion of biomolecules during stamping that may hamper the production of high quality arrays by successive stamping without re-inking. On the other hand, taking into account that the loading of the stamp is very fast and that high quality deposition by μCP of DNA molecules takes less than 15 sec to give optimal fluorescence signals, it appears more favorable to re-ink the stamp during 15 – 30 sec after each print, which is eventually faster than consecutive print.

### Comparison between μCP deposition and contact deposition using metal pins

**Figure 8:**
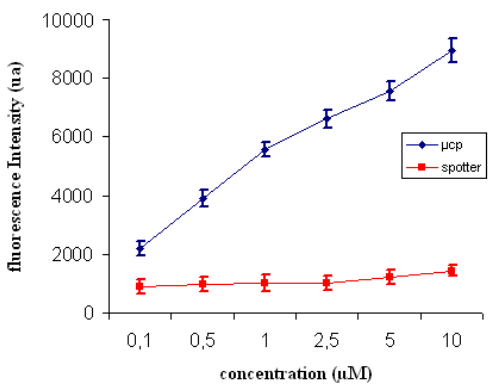
**Comparison between μCP deposition and contact deposition using metal pins**. Evolution of the fluorescence intensity in arbitrary units as a function of the concentration of the solution containing the probe molecules. 60 μm diameter spots of 20-mer oligonucleotides from *HSP12*, were deposited using a commercial Spotter (VersArray ChipWriter Pro, BIO-RAD) and then hybridized with the complementary labeled molecules. Disks and square of the same dimension were printed by μCP and treated exactly in the same conditions.

In order to compare μCP with a conventional spotting method, we performed a dedicated experiment in which the fluorescence intensity of DNA array was determined as a function of the concentration of the DNA probe used to manufacture the slides by the two techniques. To allow a direct comparison between the two methods, spots of 60 μm diameter size made with different concentration of 20-mer oligonucleotides from *HSP12 *were spotted with a commercial spotter (VersArray ChipWriter Pro, Biorad company) on a dendrislide, and disks of the same dimension were printed by μCP under the same condition. The arrays were then hybridized with the complementary labeled molecules. Fig. 8 shows the evolution of the fluorescence intensity in arbitrary units as a function of the initial concentration of the probe. From a range of 0.1 to 10 μM, the fluorescence signal was 5 to 10-fold higher when the deposition was performed by μCP than by a conventional spotter. This significant difference could be explained by the fact that deposition with a dry stamp in which the DNA molecules are delivered at the interface between the elastomeric material and the slide surface could offer uniform layers of densely packed molecules. Conversely, the deposition of a liquid droplet on the slide surface, which is let to evaporate, may give irregular layers of dispersed molecules. Alternatively or complementary to this explanation, it is possible to consider that the probes printed on the surface by μCP are better organized than by spotting, enabling a greater amount of targets accessible to the probes. In any case, for a given signal/noise ratio, the amount of probe molecules is significantly lower to get the same hybridization signals using μCP as compared to the spotting technology. This could be in the future a reasonable advantage of this technique taking into account the prohibitive price of DNA probe molecules. Moreover, this printing procedure is versatile and gives also excellent results with longer DNA molecules or double stranded PCR fragments.

### Mutation detection

**Figure 9:**
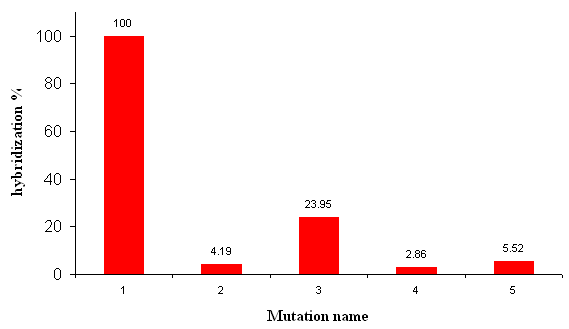
**Mutation detection**. Comparison of the hybridization signal intensity of the target molecules on 5 different printed patterns differing by only single or double mutations. "Mutation" 1 corresponds to the exact match of the target molecule and serves as a reference. Five 20-mer oligonucleotides probes were printed at 10 μM in Na-Pi buffer 0.3 M, pH 9.0 on a dendrislide. These oligonucleotides were part of the yeast *HSP12 *sequence, and varied from each other by a single or two mutations proximal to the 5' end or 3' end or in the middle of the sequence. The 20-mer sequences from *HSP12 *are noted as follows: 1: NH2 5'-AATATGTTTCCGGTCGTGTC-3'; 2: NH2 5'-AATATGTTTCAGGTCGTGTC-3'; 3: NH2 5'-AATATGTTTCCGGTCGTGTA-3'; 4: NH2 5'-AATATGATTCCGGACGTGTC-3'; 5: NH2 5'-AATAAGTTTCCGGTCGTGTC-3'; Hybridisation was carried out with Cy5-labelled oligonucleotide (Cy5 5'-GACACGACCGGAAACATATT 3'). Values of fluorescence intensity were measured at 635 nm with the GenePix 4000B from axon at 600 PMT and correspond to an average of 4 experiments. Statistics errors are less than 0.4% for the 4 experiments.

Having demonstrated that oligonucleotides can be successfully printed in multiple copies, yielding uniform patterns, we investigated the possibility to manufacture an array bearing short oligonucleotides of a given gene by μCP for detecting a single mutation as it can be made with the DNA microarray technology [11,12]. We printed 5 different 20-mer oligonucleotides from *HSP12*, encoding a protein chaperone in yeast [13]. These probes differed from each other by a single or a double base mutation at positions proximal to the 5' or 3' end or in the middle of the sequence. These oligonucleotides were then hybridized with Cy5-labelled cDNA prepared from total yeast RNA (see method section for additional details) in the automatic hybridization room. We compared the hybridization intensity of the target molecules on the printed patterns with that from the perfectly matching target sequence to the 20-mer oligonucleotide probe. We observed that whatever the position and nature of the mutation, the hybridization signal was considerably reduced for mutated sequences. As expected, the position of the mutation along the sequence of the probe molecule strongly influenced the hybridization ratio (Fig 9). This experiment was repeated 4 times independently and yielded highly reproducible data with a statistical deviation of <1%. Altogether, these results were very similar to those obtained using microarrays fabricated with dendrislides by a conventional spotting method [7]. This indicates that the quality of the arrays printed by μCP with respect to hybridization assay is largely equivalent to arrays produced by conventional deposition techniques.

## Conclusion

In this work, we demonstrated that μCP is a new potential technology platform to pattern DNA microarrays at a relatively high speed, high resolution and high reproducibility. Two additional features which may provide significant advantages of this technology over the conventional spotting technologies are: (i) the simplicity of the μCP associated with the low cost of the material employed to make the stamp, and (ii) the arrays made by μCP technology provide 10-times higher fluorescence intensity after hybridization compare to those manufactured by conventional spotting technology. With these advantages in mind, our next step will be the fabrication of a dedicated automatic X, Y, Z controlled tool for printing different probe molecules with a high throughput. In the future, μCP may help to simplify, accelerate and improve the fabrication of microarrays and increase significantly their reliability and accessibility in *i.e. *clinical applications.

## Methods

### Stamp fabrication

The first step of fabrication consists in generating a silicon master. This was achieved by proximity U.V. photolithography on a Si [100] wafer coated with positive resist (AZ 1529), and pattern transfer by deep Reactive Ion Etching (1.4 μm deep). For submicronic patterns, Electron beam lithography on PMMA (PolyMethylMetAcrylate) was used instead of UV photolithography and the etch depth was limited to 100 nm. To enable simple demoulding of this master, an anti-adhesive treatment is carried out using silanisation in liquid phase with OTS (octadecyltrichlorosilane). The final step consists to cure the PDMS pre-polymer solution containing a mixture (10:1 mass ratio) of PDMS oligomers and a reticular agent from Sylgard 184 Kit (Dow Corning) on the silicon master. The PDMS was thermally cured at 120°C for 90 min or for 12 hr at 80°C (both methods giving similar results of stamping). A silicon master can be reused more than 50 times and each stamp can be used for a large number of prints (>100).

### Surface chemistry of the substrate

Two kinds of microscope glass slides were used for spotting and printing the probes. Using "electrostatic" glass slides that are positively charged amine glass slides (Ultra Gap, Dow corning), the printed/spotted probes were cross-linked onto the amine surface by UV light at 300 mJ. With dendrislides (home made slide bearing generation 4 dendrimers, see [7], and our web site: ), a covalent attachment of the probes on the glass surface through aldehyde function of the dendrimers was performed [8,9]). After spotting, the dendrislides were allowed to dry overnight at room temperature. The reduction of the imines function formed between probes and dendrimer was carried out by immersion of the slides into a solution containing NaBH_4 _at 3.5 mg/ml for 3 hr at room temperature under agitation. The DNA slides were washed three times in water during 2 min, at room temperature and then dried under a stream of nitrogen.

### Stamping process

Stamps were incubated with 2–20 μl of a 10 μM oligonucleotide solution made in Na-phosphate buffer 0.3 M, pH 9 for only 30 sec (unless mentioned differently), and then blown dried under a stream of nitrogen. Then, the stamp was printed manually onto the substrate surface and left in place during a controlled contact time. A 35-mer 5'-labelled Cy5-oligonucleotide-3'NH_2 _(5'Cy5-TTAGCGCATTTTGGCATATTTGGGCGGACAACTT-NH_2_-3'), a 35-mer 5'-amino modified (5'NH_2_-GTGATCGTTGTATCGAGGAATACTCCGATACCATT) and 70-mer 5'NH_2 _oligonucleotides corresponding to yeast *HSP12 *gene (from Qiagen/Operon yeast set) were used in spotting and printing experiments. The PCR fragment was a 500 bp amplified fragment on *HSP12 *gene using universal primers as described elsewhere [7].

### Preparation of labeled targets

The target was a 15-mer 5'-labelled Cy5 oligonucleotide (Cy5-AATGGTATCGGAGTA) complementary to the 35-mer probes (5'NH_2_-GTGATCGTTGTATCGAGGAATACTCCGATACCATT). Other targets were prepared from total yeast RNA as a template by incorporation of fluorescent-labeled Cy5 or Cy3-dCTP during first-stand cDNA synthesis. The labeling reaction and cDNA purification was carried out with 15 μg total RNA using the LabelStar Kit from Qiagen following the manufacturer's instruction.

### Hybridization

In initial experiments, the hybridization was carried out in an hybridization cassette (Corning Inc), according to the standard protocol used in the lab for microarray technology  in the presence of 20 μl solution containing 16.5 μl Dig Easy buffer (Roche Diagnostic), 1 μl of denatured salmon sperm DNA and 2.5 μl of labeled target and covered with a 2.2 cm^2 ^cover slip to achieve a uniformed hybridization reaction during 15 min. After hybridization, the slides were washed for 2 min in 2 × SSC/0.1% (v/v) SDS; 2 min in 0.2 × SSC/0.1% (v/v) SDS and 2 min in 0.2 SSC at room temperature, and then dried under a nitrogen stream. In experiments reported on Figure 8, hybridization was carried out with an automatic hybridization room (Discovery from Ventana Medical System, Inc). Prehybridization was carried out with a freshly prepared solution of 1% BSA, 2 × SSC, 0.2% SDS during 1 h 30 at 42°C. After automatic washing according to manufacturer instruction, the slides were hybridized for 8 hr in a 200 μL of ChipHybeTM buffer (Ventana Medical System, Inc) containing 20 μl of labeled and purified cDNA.Fluorescence imaging. Fluorescent images were captured with the laser scanner GenePix 4000 B from Axon at appropriate sensitivity levels of photomultiplier (PMT). The scanner run and collects data in 5 μm steps, then averages the data into 10 μm pixels. For correct data treatment, only features bigger than 10 μm were used.

## Authors' contributions

C.T. and V.L carried out the technological and biological part of the work and wrote the first draft of the manuscript. E.T. carried out the chemical part of the study. JF and CV conceived of the study, participated in the design of the experiments, and finalized the writing of the manuscript. All authors read and approved the final manuscript.
